# Maternal transmission disequilibrium of rs2248359 in type 2 diabetes mellitus families and its association with vitamin D level in offspring

**DOI:** 10.1038/s41598-018-19838-5

**Published:** 2018-01-22

**Authors:** Songcheng Yu, Xing Li, Yan Wang, Zhenxing Mao, Chongjian Wang, Yue Ba, Wenjie Li

**Affiliations:** College of Public Health, Zhengzhou University, Zhengzhou, 450001 China

## Abstract

Association between T2DM and vitamin D deficiency has been reported in many epidemiologic studies. 24-hydroxylase encoded by *CYP24A1* is the enzyme that degrades the active vitamin D metabolite. Variation in *CYP24A1* may be associated with T2DM. This study investigates the association between rs2248359 in *CYP24A1* and T2DM by a family-based association test (FBAT) and in a case-control study. The FBAT results revealed that there was transmission disequilibrium for allele T in both additive model (*Z* = 2.041, *P* = 0.041227) and dominant model (*Z* = 2.722, *P* = 0.006496). Results of the case-control study suggested that rs2248359 may be a risk factor for female T2DM (*P* = 0.036) but not for male T2DM (*P* = 0.816). Furthermore, excessive transmission of allele T in T2DM offspring was observed compared with the non-T2DM offspring (*OR* 1.392; 95%*CI* 1.024–1.894; *P* = 0.035). In addition, combination of maternal CT and paternal CC genotypes had significant synergistic effect on obtaining CT genotype for offspring with T2DM (*OR* 6.245; 95%*CI* 1.868–20.883; *P* = 0.004). Besides, lower level of 25(OH)D in T2DM offspring with genotype CT was observed as compared with the non-T2DM offspring (*P* = 0.013). These data suggest that maternal transmission disequilibrium of allele T may be a risk factor for T2DM and vitamin D deficiency in T2DM offspring.

## Introduction

Type 2 diabetes mellitus (T2DM) is a metabolic disease resulting from insulin resistance, defects in insulin secretion or both, accounting for 90% diabetes^1,2^. The data from International Diabetes Federal (IDF) revealed that 415 million people all over the world are suffering from diabetes. This figure is estimated to rise up to 642 million by 2040. It has been estimated that one person would die due to diabetes every 6 seconds^3^. According to IDF statistics, about 673–1197 billion dollars was spent on global treatment and prevention of diabetes in 2015. It would reach 802–1452 billion dollars by 2040^3^. Thus, diabetes can be a great burden to individuals and the society. Primary prevention of T2DM is a global public health imperative.

It is well known that vitamin D plays an important role in calcium homeostasis and bone metabolism^4^. More and more evidence suggest that vitamin D can be a endocrine substance to exert other biological activities, such as proliferation, differentiation, apoptosis, and immune response, etc.^5^. Thus, vitamin D deficiency and insufficiency may be risk factors for a wide spectrum of acute and chronic illnesses^6,7^. In the past decade, many epidemiologic studies have reported an association between T2DM and vitamin D deficiency^8,9^. Vitamin D plays an important role in beta cell function, insulin sensitivity and systemic inflammation, which implies that vitamin D deficiency might be a possible pathway in the development of T2DM^10^.

Vitamin D itself does not have the biological function mentioned above^11^. Two successive hydroxylations are required to form the active hormone metabolite 1,25- dihydroxy vitamin D (1,25(OH)_2_D) to exert its biological actions via binding the vitamin D receptor. The first one is catalyzed by 25-hydroxylase in the liver to produce 25-hydroxy vitamin D (25(OH)D). The other one is catalyzed by 1α-hydroxylase in the kidney at the C_1_ position to form 1,25(OH)_2_D^12^. The active metabolite 1,25(OH)_2_D is then degraded by 24-hydroxylase encoded by *CYP24A1* to the water soluble and biologically inactive excretory product: calcitoic acid^13^. It has been reported that *CYP24A1* plays an important role to maintain the proper level of 1,25(OH)_2_D *in vivo*^14^. Therefore, abnormal expression of *CYP24A1* may be associated with vitamin D related diseases.

rs2248359, located at the promotor region of *CYP24A1*, has been shown to affect *CYP24A1* expression and to be associated with multiple diseases. Adaikalavan Ramasamy found that rs2248359 was strongly associated with increased expression of *CYP24A1* in frontal cortex^15^. It has been reported that the allele C of rs2248359 was related to an increased risk of multiple sclerosis^16^. In addition, a number of studies have shown that rs2248359 was associated with asthma and atopic dermatitis^17,18^. Besides, it has been reported that rs2248359 was associated with obesity in Chinese women^19^. Thus, rs2248359 may be associated with vitamin D related diseases by regulating the expression of *CYP24A1*. However, up to date, no study reported its association with T2DM. In this study, we investigate the relationship between rs2248359 and T2DM, as well as vitamin D level by a family-based association test and in a case-control study. Furthermore, we want to find out whether there is an association between rs2248359 and 25(OH)D level in T2DM offspring. The results will shed light on the occurrence, development and prevention of T2DM.

## Results

### Subject characteristics

A total of 1560 participants from 419 pedigrees were included in this study. On one hand, there were 280 T2DM patients from 241 pedigrees. Both father and mother were found for 24 T2DM patients. Either father or mother was found for another 33 T2DM patients. And 210 T2DM patients had offspring. On the other hand, 178 pedigrees had no T2DM patients. 27 pedigrees had T2DM history. And 99 couples from 99 different pedigrees without T2DM history had their offspring. The characteristics of the participants were summarized in Table 1.

**Table 1:** Demographic characteristic of the study participants.

Variable		T2DM pedigree (N = 897)		Non-T2DM pedigree (N = 663)
		Non-T2DM	T2DM	
Male (%)		312 (50.6)	108 (38.6)	341 (51.4)
Age (years)		44.8 ± 19.2	59.4 ± 12.5	48.1 ± 19.5
Smoking (%)	Never	290 (47.0)	157 (55.7)	329 (49.6)
	Ever	34 (5.5)	20 (7.1)	37 (5.6)
	Current	164 (26.6)	47 (16.8)	182 (27.5)
	Passive	129 (20.9)	57 (20.4)	115 (17.3)
Drinking (%)	Never	479 (77.6)	243 (86.8)	496 (74.8)
	Ever	44 (7.1)	15 (5.4)	58 (8.7)
	Current	94 (15.2)	22 (7.9)	109 (16.4)
High fat diet intake (%)		88 (14.3)	34 (12.1)	112 (16.9)
Vegetables (%)		172 (27.9)	56 (20)	195 (29.4)
Physical activity (%)	Low	215 (34.8)	138 (49.3)	252 (38.1)
	moderate	135 (21.9)	50 (17.9)	118 (17.8)
	High	267 (43.3)	92 (32.9)	293 (44.2)
Family history of T2DM (%)	Yes	271 (43.9)	60 (21.4)	30 (4.5)
BMI (kg/m^2^)		24.6 ± 4.4	26.5 ± 3.8	24.5 ± 4.2

### Family-based association test

Family-based association test (xxx) was conducted to examine whether there is an association between rs2248359 and T2DM. As shown in Table 2, in the additive model, increased transmission of allele T was found in 16 informative families (*Z* = 2.041, *P* = 0.041227). In the dominant model, excessive transmission of allele T was also found in 16 informative families (*Z* = 2.722, *P* = 0.006496). These results suggest that there is transmission disequilibrium for allele T in T2DM families.

**Table 2:** The results of family-based association test between rs2248359 and T2DM.

Model	Allele	afreq	Fam#	S-E(S)	Var(S)	*Z*	*P*
Additive	C	0.617	16	−5	6	−2.041	0.041227
	T	0.383	16	5	6	2.041	0.041227*
Dominant	T	0.383	13	5	3.375	2.722	0.006496*
Recessive	C	0.617	13	−5	3.375	−2.722	0.006496

Note: 232 pedigrees containing 280 T2DM patients were read in FBAT software. S-E(S) and Var (S) are the expected value and variance of the test statistic. *Z*: the test statistic; *P*: significance level. *The significant association between allele and T2DM (*Z* > 0 and *P* < 0.05).

### rs2248359 variation in T2DM patients and their offspring

In this study, 210 cases of the 280 T2DM patients had offspring. The T2DM prevalence among offspring of T2DM patients was 9.2%. Using 99 nuclear triad families without T2DM history as control, genotype distributions of rs2248359 for male (father), female (mother) and their offspring were compared respectively. The average age of offspring of T2DM and non-T2DM were 36.1 ± 13.2 and 31.0 ± 12.3, respectively. The male/female ratios of the offspring of T2DM and non-T2DM were 1.26 and 2.17, respectively. The rs2248359 variations were displayed in Table 3. All the observed genotype distributions were comparable with Hardy-Weinberg Equilibrium in both T2DM families and non-T2DM families (*P* > 0.05).There was no significant difference in genotype and allele frequencies between male T2DM patients and the controls (*P* = 0.816). However, difference was found for genotype in female T2DM patients and female non-T2DM (*P* = 0.036). When the dominant model for allele T was applied (CT + TT vs CC), there was about 2 folds increased risk for T2DM (*OR* 1.964; 95% *CI* 1.138–3.389; *P* = 0.015). And heterozygous genotype (CT vs CC + TT) also has an increased risk for T2DM (*OR* 1.912; 95% *CI* 1.127–3.244; *P* = 0.016). On the other hand, significant difference in genotype and allele frequencies between offspring of T2DM and non-T2DM was found. Excessive amount of allele T was observed in T2DM offspring (*OR* 1.392; 95% *CI* 1.024–1.894; *P* = 0.035). And allele T has increased risk for T2DM offspring in the dominant model (CT + TT vs CC; *OR* 1.710; 95% *CI* 1.121–2.608; *P* = 0.012). These data indicate that allele T of rs2248359 is susceptible for female T2DM and T2DM offspring.

**Table 3:** rs2248359 variation in T2DM family and non-T2DM family.

Genotype/Allele	Father			Mother			Offspring			
	non-T2DM(N = 97)	T2DM(N = 77)	*P*	non-T2DM(N = 98)	T2DM(N = 133)	*P*	non-T2DM(N = 127)	T2DM(N = 303)		*P*
CC	41	29	0.816	44	39	0.036^*#^	59	102		0.044^*#^
CT	40	35		41	77		52	155		
TT	16	13		13	17		16	46		
C	122	93	0.634	129	155	0.100	170	359		0.035*
T	72	61		67	111		84	247		
*OR* (95% *CI*)										
CT/TT vs CC	1.212(0.657–2.235)		0.538	1.964(1.138–3.389)		0.015*	1.710(1.121–2.608)			0.012*
TT vs CC/CT	1.028 (0.461–2.293)		0.946	0.958(0.442–2.079)		0.914	1.242(0.674–2.287)			0.487
CT vs CC/TT	1.188(0.649–2.172)		0.577	1.912 (1.127–3.244)		0.016*	1.511(0.993–2.297)			0.053
T vs C	1.111(0.719–1.717)		0.634	1.379(0.940–2.022)		0.100	1.392(1.024–1.894)			0.035*

Note: 210 T2DM patients as parents and their offspring were compared with 99 non-T2DM couples and their offspring. Number for non-T2DM as father or mother was less than 99 due to missed information of genotyping. Number of offspring was larger than the sum of T2DM patients or non-T2DM parents since more than one offspring were included in some pedigrees. *P < 0.05. ^#^The *P* value is also less than 0.05 after adjustment of gender and age by logistic regression.

### Maternal-paternal-offspring genotype incompatibility of rs2248359

**Figure 1:**
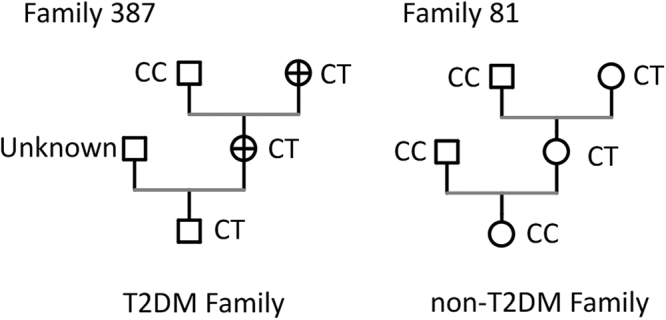
Typical pedigrees for maternal-paternal-offspring genotype incompatibility of rs2248359. Family 387 containing T2DM patients and family 81 with no T2DM history were shown as contrast. + denotes T2DM patient.

In order to evaluate the synergistic effects of maternal-paternal-offspring genotype incompatibility combinations on risk of T2DM, 24 nuclear triad families of T2DM (both father and mother were found for T2DM patient) and 97 nuclear triad families of non-T2DM (without T2DM history) were included. As shown in Table 4, when maternal and paternal genotypes were the CT and CC combination, increased genotype CT was observed in offspring with T2DM (*OR* 6.245; 95% *CI* 1.868–20.883; *P* = 0.004). And no other maternal and paternal genotype combination has significant synergistic effects (*P* > 0.05). Thus, allele T from mother had much higher transmission frequency in T2DM families if maternal and paternal genotypes were CT and CC, respectively. A typical pedigree is shown in Fig. 1.

**Table 4:** Maternal-paternal-offspring genotype incompatibility for rs2248359.

Maternal	Paternal	Offspring	T2DM(N = 24)	Non-T2DM(N = 97)	*OR*(95% *CI*)	*P*
CC	CC	CC	2	17	0.428(0.092–1.994)	0.359
CC	CT	CC	0	11	—	—
CC	CT	CT	2	7	1.169 (0.227–6.020)	1.000
CC	TT	CT	2	9	0.889(0.179–4.411)	1.000
CT	CC	CC	0	13	—	—
CT	CC	CT	7	6	6.245(1.868–20.883)	0.004*
CT	CT	CC	0	3	—	—
CT	CT	CT	3	9	1.397(0.348–5.612)	0.439
CT	CT	TT	2	5	1.673(0.304–9.198)	0.421
CT	TT	CT	2	2	4.318(0.567–32.360)	0.176
CT	TT	TT	1	2	2.065(0.179–23.772)	0.488
TT	CC	CT	2	5	1.673(0.304–9.198)	0.421
TT	CT	CT	0	3	—	—
TT	CT	TT	0	1	—	—
TT	TT	TT	1	4	1.011(0.108–9.480)	1.000

Note: 24 triad nuclear families for T2DM and 97 triad nuclear families for non-T2DM were included. When the observed frequency was zero, the *OR* value and *P* value were expressed as “-”. *P < 0.05.

### Effect of rs2248359 on level of 25(OH)D in T2DM offspring

**Figure 2:**
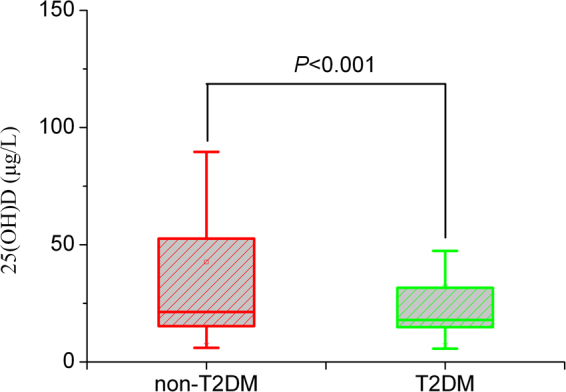
25(OH)D level in T2DM and non-T2DM. Wilcoxon rank sum test was applied to analyze the difference. Test level was *α* = 0.05.

**Figure 3:**
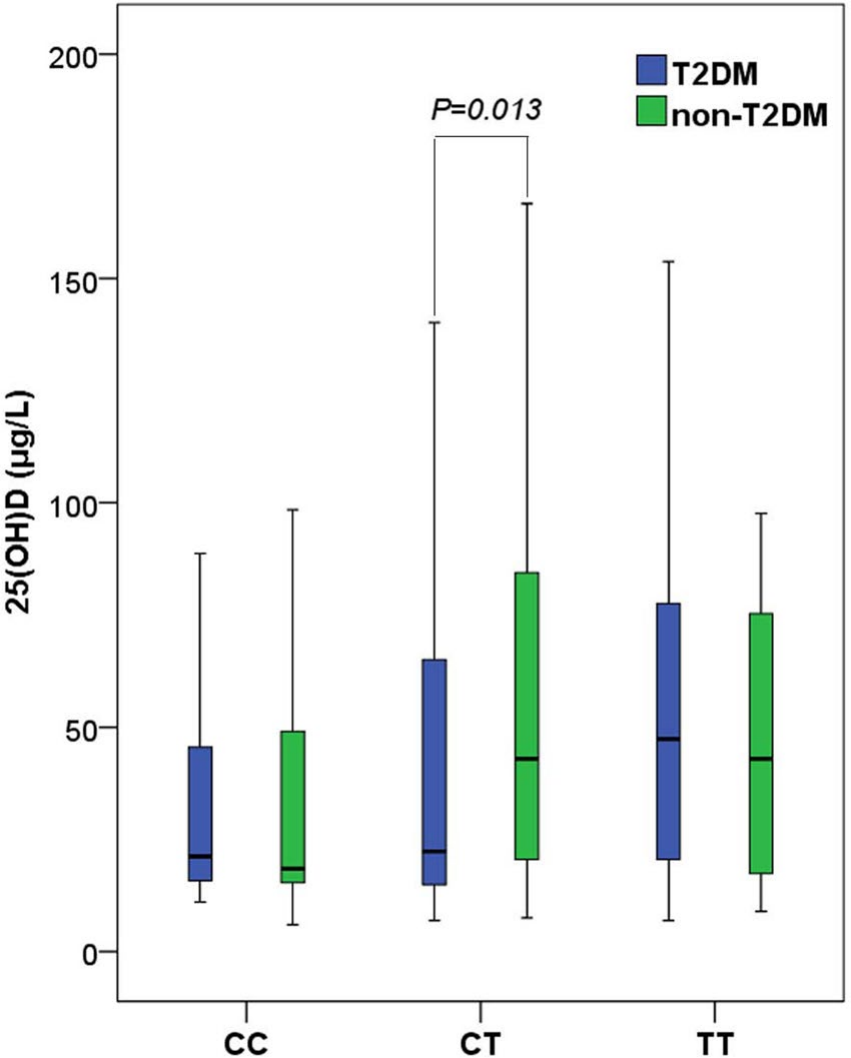
25(OH)D level for different genotypes in T2DM and non-T2DM offspring. Wilcoxon rank sum test was applied to analyze the difference. Test level was *α* = 0.05.

In accordance with reported literature^8^, association between 25(OH)D level and T2DM was also found in this study (Fig. 2). Besides, we found that T2DM offspring with genotype CT had lower level of 25(OH)D than the non-T2DM offspring (Fig. 3). No significant difference was found for CC and TT genotypes.

## Discussion

T2DM is a metabolic disease caused by both genetic and environmental factors. Vitamin D deficiency has been reported to be a risk factor for T2DM^8^. Thus, genetic variation in vitamin D metabolism-related genes may play a role in the development of T2DM^20^. *CYP24A1* encoding the enzyme to degrade the active vitamin D metabolite 1,25(OH)_2_D plays an important role in regulation of vitamin D function^13,14^. rs2248359 located in the promotor region has an effect on *CYP24A1* expression^15^. Therefore, investigation the association between rs2248359 and T2DM will shed light on molecular mechanism accounted for vitamin D. In this study, both a family-based association test and a case-control study were conducted to investigate the association between rs2248359 and T2DM. This is the first study on the transmission disequilibrium of allele T in T2DM families. And maternal-paternal-offspring genotype incompatibility was found for CT-CC-CT combination. In addition, T2DM offspring with genotype CT had lower level of 25(OH)D than non-T2DM offspring. Thus, this work provided new evidence on the association between vitamin D and T2DM.

rs2248359 may play a role in T2DM development through inflammation regulation, a major process leading to insulin resistance^21^. Y. Wang had reported that rs2248359 had an effect on inflammation. Allele T of rs2248359 has been associated with increased leukocyte counts^22^. And vitamin D was found to reduce inflammation by decreasing the release of cytokines and chemokines which drive inflammation^10^. Thus, allele T may be linked to insulin resistance, which is induced by vitamin D deficiency related inflammation.

In this study, it was allele T that showed transmission disequilibrium in T2DM families. Allele T was associated with increased risk of T2DM in both dominant and heterozygous model. And 25(OH)D level was lower in T2DM offspring with CT genotype than non-T2DM offspring. Thus, allele T may be a risk factor for vitamin D deficiency. However, allele C instead of allele T has a reverse function to vitamin D activity. Allele C of rs2248359 has been reported to increase expression of *CYP24A1* in frontal cortex^15^. Overexpression of *CYP24A1* means shorter half-life of 1,25(OH)_2_D due to a faster breakdown^14^. The result is inconsistent with the current study. Perhaps, tissue specification and noncanonical mechanism should be considered. Further experiments investigating molecular and cellular function of rs2248359 and its effect on the development of T2DM are required to confirm this hypothesis.

To our knowledge, this is the first study concerning maternal-paternal-offspring genotype incompatibility of rs2248359 in relation to T2DM risk for the offspring. In this study, we found that the combination of maternal CT and paternal CC genotypes increased the frequency of CT genotype in offspring with T2DM. Such phenomenon was not observed for the combination of paternal CT and maternal CC genotypes. These results suggest that maternal derived copy of the variant allele T may be a risk factor for T2DM offspring.

It is interesting that both dominant model of allele T (CT + TT vs CC) and heterozygous genotype (CT vs CC + TT) showed increased risk for female T2DM, not male T2DM. Gender-specific phenomenon has also been reported by others. Leif Groop and colleagues found a gender-specific paternal effect on insulin^23^. Higher age-specific rates of type 2 diabetes among offspring of women with diabetes were observed. And risk of type 2 diabetes in offspring of diabetic mothers was higher than in those of diabetic fathers^24^. Excess maternal transmission of genetic variation to offspring with type 2 diabetes was observed by Prosad *et al*.^25^. Besides, DNA methylation may be a factor that influences the gender-specific effect of allele T of rs2248359. Methylation status has been reported to be a possible mechanism for maternal effect in type 2 diabetes transmission^25–27^. The sequence near rs2248359 is as follows: CAAACATACACAATTTCTAGAGTTA[C/T]CGCGGCTGGCAAGAGTAGAACTCGC. There are two CpG islands next to rs2248359. Thus, methylation of these CpGs may play a role in the gender-specific effect of allele T of rs2248359 on T2DM found in this study.

An excessive amount of allele T was observed in T2DM offspring compared with non-T2DM offspring. And maternal CT genotype combined with paternal CC genotype was an important risk factor for T2DM offspring with CT genotype. These data also indicated that maternal allele T of rs2248359 may be a risk factor for T2DM offspring in families with T2DM history. Furthermore, lower level of 25(OH)D in T2DM offspring with genotype CT was observed as compared with offspring of non-T2DM, but not the other two genotypes (CC and TT). These results indicate that individuals with CT genotype and T2DM parents may be more likely to suffer from vitamin D deficiency and T2DM.

New light was shed on the association between T2DM and vitamin D metabolism-related gene in this work. However, there are also limitations. One of the limitations of this study is small sample size. Although 419 pedigrees with 280 T2DM patients were included in this study, both father and mother were found for only 24 T2DM patients. Either father or mother was found for another 33 T2DM patients. As the average age of T2DM patients was 59.4 years, most of their parents had passed away. It is a great difficulty to collect the information of a triad nuclear family for T2DM patient in a cross-sectional study. Therefore, cohort studies with larger sample size for T2DM families are needed to verify the findings in this study. Furthermore, data of *CYP24A1* expression was lacking in this study. Family-based association was found between rs2248359 and T2DM. And CT genotype was linked to lower 25(OH)D level in T2DM offspring. rs2248359 located in the promotor region of *CPY24A1* gene may influence the expression. But association between rs2248359 and *CPY24A1* expression was not investigated in this study. Further research on this aspect is warranted. In addition, lower level of 25(OH)D in T2DM offspring with genotype CT was observed as compared with the offspring of non-T2DM, but the variation of 25(OH)D level among the participants is considerable. Large individual variation in this study may lead to less reliable results. Nevertheless, the difference of 25(OH)D levels between T2DM offspring and non-T2DM offspring was contrasted by Wilcoxon rank sum test. And the sample was analyzed in a certificated third-party medical laboratory, the result is relatively reliable. A more reliable study is needed to confirm this finding.

To sum up, there is transmission disequilibrium for allele T of rs2248359 in T2DM family. Maternal derived copy of the variant allele T is the principal risk factor for T2DM offspring. Individuals with CT genotype and T2DM parents may be more likely to suffer from vitamin D deficiency. These results are valuable for T2DM prevention and vitamin D supplementation guidance.

## Methods

### Study subjects

According to cluster sampling design, a family-based representative sample of 1560 participants from 419 pedigrees was recruited in Qiaomiao town, Wuzhi County, Henan province of China in 2013. Information about education, diet, lifestyle, family history of diabetes, etc. was obtained by face-to-face interview. Fasting venous blood samples were collected for biochemical determination and DNA extraction. Diagnosis of T2DM was based on the criteria of the World Health Organization (1999) and the guidelines of American Diabetes Association (2002). And all the non-T2DM had normal glucose tolerance by an oral glucose tolerance test.

This study was in accordance with the Code of Ethics of the World Medical Association and approved by Zhengzhou University Medical Ethics Committee. Written informed consent was signed by all the participants. And their privacy information was protected.

### Measurement of 25(OH)D

The concentrations of serum 25(OH)D were determined with electrochemical luminescence in a certificated third-party medical laboratory of Kingmed Center for Clinical Co., Ltd.

### DNA extraction

DNA blood kit from Bioteke Corporation (Beijing, China) was applied for genomic DNA extraction from peripheral blood. All the operations were consistent with the standard procedures provided by the manufacturer. The quality and quantity of the extracted DNA samples were examined with Nanodrop 2000 spectrophotometer (Thermo Fisher Scientific, US)^28^. Ratio value of OD_260_/OD_280_ larger than 1.7 was set as quality criterion.

### Genotyping

Based on the GWAS results from Chinese women, rs2248359 located in the promotor region of *CYP24A1* was selected in this study^19^. The TaqMan® SNP genotyping assays in 7500 fast system (Applied Biosystems, California, US) was applied for genotyping work. Briefly, TaqMan® Predesigned SNP Genotyping Assay for rs2248359 (Assay ID: C__16261116_10) and TaqMan® Genotyping Master Mix (Catalog No. 4371355) was purchased from Applied Biosystems. The reaction solution was prepared by adding 10 μL Master Mix, 1 μL Genotyping Assay, 0.5 μL DNA sample and 8.5 μL nuclease-free water. The reaction condition was as follows: 95 °C for 10 min, followed by 40 cycles of 95 °C for 15 s and 60 °C for 1 min. The genotyping results were determined automatically by the system software.

### Statistical Analysis

We analyzed the data by following strategies. First, the family-based association test (FBAT) software (V2.0.4Q, https://www.hsph.harvard.edu/fbat/fbat.htm) was applied to examine the association between rs2248359 and T2DM. Second, distributions of rs2248359 genotype in T2DM families and non-T2DM families without T2DM history were examined with *Chi-square* test. Third, the maternal-paternal-offspring genotype incompatibility of rs2248359 was studied by *Chi-square* test. Finally, the difference of 25(OH)D levels between different T2DM offspring and non-T2DM offspring was contrasted by Wilcoxon rank sum test, due to its non-normal distribution. All the statistical analysis except FBAT was conducted with SPSS 21.0 (IBM SPSS, New York, US). The statistical tests were two-sided and the test criterion was set as α = 0.05.

### Data availability

All data generated or analyzed during this study are included in this published article.

### What is already known on this topic?

Association between T2DM and vitamin D deficiency has been reported in many epidemiologic studies. The active vitamin D metabolite 1,25(OH)_2_D is degraded by 24-hydroxylase encoded by *CYP24A1* to water soluble and biologically inactive excretory products. rs2248359, located in the promotor region of *CYP24A1*, has been shown to affect *CYP24A1* expression and associated with vitamin D deficiency related diseases. However, no study has reported its association with T2DM.

### What does this study add?

The family-based association test results revealed that there was transmission disequilibrium for allele T of rs2248359 in T2DM families. The combination of maternal CT and paternal CC genotypes had significant synergistic effect on obtaining CT genotype among offspring in T2DM families. Furthermore, offspring of T2DM with CT genotype had lower level of 25(OH)D than the offspring of non-T2DM. Thus, maternal transmission disequilibrium of allele T may be a risk factor for lower level of vitamin D in T2DM offspring. These results are valuable for T2DM prevention and vitamin D supplementation guidance.
